# Oral Midazolam for Voiding Dysfunction in Children Undergoing Voiding Cystourethrography: A Controlled Randomized Clinical Trial

**DOI:** 10.5812/numonthly.17168

**Published:** 2014-05-01

**Authors:** Anoush Azarfar, Mohammad Esmaeeili, Azadeh Farrokh, Ali Alamdaran, Aghilallah Keykhosravi, Mahboobe Neamatshahi, Alireza Hebrani, Yalda Ravanshad

**Affiliations:** 1 Department of Pediatric, School of Medicine, Mashhad University of Medical Sciences, Mashhad, IR Iran; 2 Department of Radiology, Mashhad University of Medical Sciences, Mashhad, IR Iran; 3 Department of Pediatric, Vaseei Hospital, Sabzevar University of Medical Sciences, Sabzevar, IR Iran; 4 Department of Social Medicine, Mashhad University of Medical Sciences, Mashhad, IR Iran; 5 Education Development Center, Mashhad University of Medical Sciences, Mashhad, IR Iran

**Keywords:** Midazolam, Voiding Cystourethrography, Child

## Abstract

**Background:**

Voiding Cystourethrography (VCUG) is the gold standard of detecting and grading the vesicoureteral reflux. Moreover, VCUG is a part of the standard review for infants and children with a urinary tract infection and urinary dysfunction.

**Objectives:**

The purpose of our study was to compare using oral midazolam in contrast to prescribing no sedative medication for voiding dysfunction in children undergoing VCUG.

**Patients and Methods:**

In a clinical trial, we studied 84 children referred for VCUG. Children were allocated randomly into two equal groups. The intervention group received 0.5 mg/kg midazolam orally half an hour before the VCUG procedure. Then both groups were compared using statistical methods.

**Results:**

Then both groups were compared using statistical methods. In more than half of the patients, the main cause of performing VCUG was urinary tract infection. Dysuria was evaluated immediately after VCUG and was more frequent in girls than in boys (P = 0.006). After one week, the urinary irritation and restlessness in the intervention group was significantly lower than the control group.

**Conclusion:**

The use of midazolam 0.5 mg/kg reduced children's stress and increased their cooperation during the procedure.

## 1. Background

Imaging of the urinary tract and kidneys has a key role in the evaluation of these organs. Nowadays there are different types of imaging methods with some advantages and disadvantages (1). Voiding cystourethrography (VCUG) is the gold standard of detecting and grading the vesicoureteral reflux (VUR). In addition to reviewing urethra (especially in boys), information on the performance and coordination of bladder and urethral sphincter can be obtained. Therefore, VCUG is a part of the standard review for infants and children with a urinary tract infection (UTI) and urinary dysfunction (2, 3). VCUG does not require a specific facility; however, due to fear and worries of the family and the patient, the medical team should talk with them prior to performing VCUG. Furthermore, a small booklet containing information on how the test is done should be accessible for the family members. In cases where children have a severe phobia, oral or intranasal midazolam can be prescribed to reduce the patients' anxiety (0.5 mg/kg orally or 0.2 mg/kg nasally). The drug is safe and effective. In addition, the presence of children parents and the use of a water-soluble anesthetic gel makes it easier (3). Although the side effects of VCUG are not common, they are important. These complications that can occur in both sexes include UTI, hematuria, cystitis as well as urinary dysfunction following a catheterization, phobia of urination, nocturia, and stopping urination (4). In the literature, psychological trauma resulting from VCUG was considered the same as from a violent rape, especially in girls (5). Herd et al. performed a clinical trial study and noted that performing VCUG for children was a stressful situation and the use of sedative drugs such as midazolam could effectively decrease the agitation and stress in children (6). Midazolam, as a sedative, did not disturb the voiding mechanism of children and did not cause any misinterpretation of the VCUG result. They concluded that using midazolam as a sedative in children could reduce stress and improve the efficiency of the process. Moreover, they showed that there was a significant difference in the stress level between those who received and those who did not receive midazolam. In another follow-up study by Herd et al., they reviewed about 17 articles on the use of sedatives before VCUG; they concluded that the use of midazolam was very effective in reducing stress in children and this drug can be prescribed without causing significant interference with the VCUG (7). In another study by Sorkhi et al., the effects of oral midazolam before the VCUG in children were evaluated. In this clinical trial that included 98 patients, different factors were assessed and they strongly recommended oral midazolam before the procedure (8). However, in most of the previous works, they have focused on the urinary dysfunction during the VCUG and paid less attention to the potential problems after the procedure in a longer period.

## 2. Objectives

The purpose of our study was to assess prescription of oral midazolam for voiding dysfunction in children undergoing VCUG, both during and days after the procedure.

## 3. Patients and Methods

This study was a clinical trial study with code IRCT201110257892. In this study, we evaluated 84 children admitted to Mashhad Children Hospital from March 2012 till March 2013 for VCUG, randomly divided into two groups. After obtaining informed consent from the parents, the intervention group received 0.5 mg/kg midazolam orally half an hour before the VCUG procedure. During VCUG, the patients were evaluated for the possibility of voiding during VCUG, restlessness, and stress. A week later, the patients were re-examined and evaluated of disorders of the urinary tract and voiding dysfunction. In the control group, the process was done without administering midazolam. When the parents did not return for follow-up, they were contacted again and the patient was dropped from the list if they did not come back. Then both groups were compared using statistical methods.

## 4. Results

**Figure 1.**
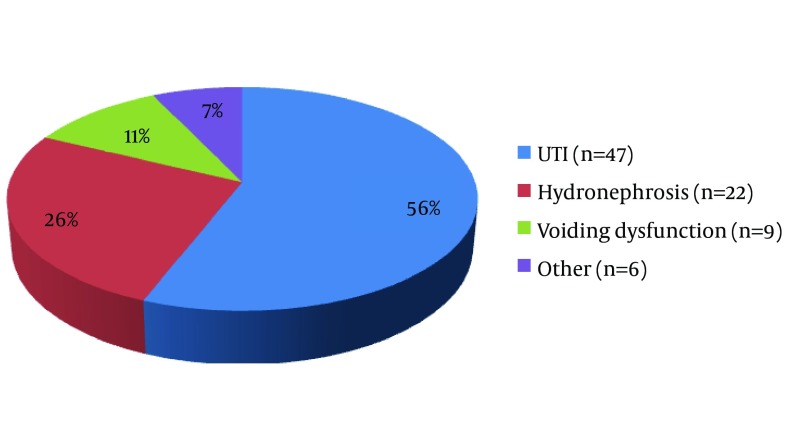
The Indications of Voiding Cystourethrography in Participants.

The mean age of patients was 6.8 ± 1.85 years. Among patients, 24 (28.6%) were boys and 60 (71.4%) were girls. As illustrated in Figure 1, the main cause of performing VCUG was UTI in more than half of the patients.

The rates of urinary incontinence (P = 0.808) and urinary frequency (P = 1) were the same between girls and boys (Table 1). Coordination in VCUG, dysuria after VCUG, urinary incontinence and frequency were significantly different in two groups but voiding dysfunction was similar (Table 2). Urinary irritation and restlessness, followed by VCUG, were significantly lower in the control group than in the intervention group (P = 0.001). However, after one week, the urinary irritation and restlessness in the intervention group was significantly lower than controls (Table 3).

**Table 1.** Urination Problems After Voiding Cystourethrography in the Intervention and Control Groups ^a^

Variable	Intervention and Control Group				P Value
	Male (n = 24)		Female (n =60)		
**Dysuria**	16 (66.66)	24	19 (40)	60	0.005
**Urinary incontinence**	5 (20.84)	24	35 (58.33)	60	0.808
**Urinary frequency**	11 (45.84)	24	28 (58.33)	60	1
**Dysuria after one week**	15 (62.5)	24	24 (40)	60	0.099

^a^ Data are presented as No. (%).

**Table 2.** Comparing Intervention and Control Groups During and Immediately After Voiding Cystourethrography ^a^

Variable	Intervention Group	Control Group	P Value
**Coordination in VCUG** ^b^			0.001
Acceptable	28 (66.7)	6 (14.3)	
Moderate	6 (14.3)	21 (50)	
Poor	8 (19)	15 (35.7)	
**Voiding dysfunction**	31 (63.8)	9 (21.4)	0.611
**No dysuria after VCUG **	29 (79)	6 (14.2)	0.001
**No VUR**	23 (54.8)	21 (50)	0.580
**No urinary incontinence and frequency**	26 (62)	12 (28.6)	0.001

^a^ Data are presented as No. (%).

^b^ Abbreviations: VCUG, voiding cystourethrography; VUR, vesicoureteral reflux.

**Table 3.** Comparison of Absence of Urinary Problems One Week after the Voiding Cystourethrography Between the Intervention and the Control Groups ^a^

Variable	Intervention Group	Control Group	P Value
**Dysuria**	29 (69)	9 (11.4)	0.001
**Urinary incontinence**	26 (62)	24 (57.2)	0.652
**Avoid of voiding**	30 (71.4)	30 (71.4)	0.575
**Urinary frequency**	26 (62)	12 (28.6)	0.001

^a^ Data are presented as No. (%).

## 5. Discussion

Imaging of kidney and urinary tract has a significant role in the diagnosis of many urinary diseases. VCUG is the gold standard of diagnosis and grading VUR. However, it can be associated with tension and uneasiness in some children. In addition to educating parents, drugs such as midazolam can be used in such cases (9). In this study, we discussed the effects of oral midazolam in patients who underwent VCUG. According to Figure 1, the most common reason to perform VCUG in children was UTI, which is consistent with the results by Sorkhi et al. (8). In our study, as illustrated in Table 1, the majority of the participants were girls. Dysuria was evaluated immediately after VCUG and was more frequent in girls than in boys (P = 0. 006); however, there was no difference after one week (P = 0. 099). The rates of urinary incontinence (P = 0.808) and urinary frequency (P = 1) were the same between girls and boys. Previous studies did not investigate these problems with regard to the gender of the patients. In our study coordination in VCUG, dysuria after VCUG, urinary incontinence and frequency had a significant difference in two groups but voiding dysfunction was almost the same (Table 2). According to Table 3, urinary irritation and restlessness, followed by VCUG, were significantly lower in the control group than in the intervention group (P = 0.001). However, after one week, the urinary irritation and restlessness in the intervention group was significantly lower than controls (Table 3), while incontinence and refusing to urinate did not differ significantly between the two groups (P = 0.652 and P = 0.575, respectively).

In his review study, Herd found that using a combination of midazolam and analgesic was useful in reducing stress in children during VCUG (7). In a study by Stockland et al., it was reported that children who were treated with midazolam experienced less stress in comparison to the children treated with placebo. The study pointed out that the majority of children in the midazolam group had an easily voiding. Finally, they concluded that in such cases, the use of midazolam for VCUG was safe (10). The work by Merguerian PA et al. showed that the sedative effect of oral midazolam had no negative effects on VCUG in detecting VUR (11). Performing VCUG in children is usually accompanied with infant irritability, restlessness, and stress that may interfere with the process. According our observation, we can conclude that using midazolam 0.5 mg/kg can reduce children's stress and increase their cooperation during the procedure. Thus, the routine use of oral midazolam is recommended for any investigating procedure including VCUG in children older than five years. In the present study, a standardized grading system to assess stress in children was not used, which is recommended for future studies.
